# Current evidence of MTX efficacy in childhood chronic uveitis: a systematic review and meta-analysis

**DOI:** 10.1186/1546-0096-9-S1-P227

**Published:** 2011-09-14

**Authors:** G Simonini, P Paudyal, G Jones, R Cimaz, GJ Macfarlane

**Affiliations:** 1 Rheumatology Unit-Department of Paediatrics, University of Florence, Italy; 2 Epidemiology Group, Institute of Applied Health Sciences, University of Aberdeen, UK

## Aim

To summarize evidence regarding the effectiveness of Methotrexate (MTX) in the treatment of childhood autoimmune chronic uveitis.

## Methods

A systematic search of articles published between January 1990 and February 2011 was conducted using following electronic databases: EMBASE, Ovid MEDLINE, EBM Reviewers–ACP Journal Club, all Cochrane library, and EBM Reviews–Database of Abstracts of Reviews of effects. Reference list and citations of those identified were also screened to find out relevant papers. Studies investigating the efficacy of MTX, as a single immunosuppressant medication in the treatment of chronic autoimmune uveitis, refractory to therapy with topic treatment and/or systemic treatment, in children (≤16 yrs) were eligible for inclusion. Primary outcome measure was the improvement of intraocular inflammation as Tyndall, as defined by the SUN working group criteria. When reported and available, tapering and/or stopping systemic steroid administration, improvement in visual acuity post MTX treatment, discontinuation of MTX, time for remission, time on remission, and safety of treatment were also considered as additional secondary additional outcomes. We determined a combined estimate of the proportion of children in the eligible studies responding to MTX. The effect measure for each study was therefore the proportion of people classified as responders.

## Results

Fifty-two potential eligible articles were selected for detailed review of the 246 found by the initial computerized search and then scrutinized applying the selection criteria. A total of 9 articles were strictly eligible and remained in the analysis. All of the included studies were retrospective chart reviews. Number of children in these studies ranged from 3 to 25 and the dose of MTX varied from 7.5 mg/mq^2^ to 30 mg/mq^2^. Altogether, 75 children out 135 included responded to the treatment. The pooled analysis suggested that MTX has favourable effect in the improvement of intraocular inflammation: 0.73 (95% CI: 0.66-0.81).

## Conclusion

The available evidence supports the use of MTX in the treatment of childhood chronic uveitis: patients on MTX can expect a 73% probability of improvement in intraocular inflammation; however randomised controlled trials on MTX are necessary before a firm conclusion can be drawn.

